# Supplementary material for: CNN_FunBar: Advanced Learning Technique for Fungi ITS Region Classification
Source: Genes (Basel). 2023 Mar 3;14(3):634. doi: 10.3390/genes14030634 (PMC10048311; doi:10.3390/genes14030634)
Supplement: Supplementary file 1 [file genes-14-00634-s001.zip › Table S1.pdf]

Table S1. **Classification performances of CNN model with different *k* values for balanced datasets at all taxonomic levels. Values of performance metrices are obtained from corresponding test datasets**

Taxonomic Level	*k*	Total no. of categories	Seq per category	Avg. Accuracy (%)	Avg. Precision (%)	Avg. Recall (%)	Avg. F1 Score (%)	Avg. MCC Score (%)
Species	4	2,752	20	84.7	85.72	84.7	83.54	84.69
		939	50	86.69	87.96	86.68	86.34	86.68
		429	100	89.14	89.78	89.13	89.01	89.11
		148	250	89.65	89.89	89.65	89.6	89.58
		117	300	88.6	88.84	88.6	88.51	88.51
		64	500	93.03	93.09	93.03	92.96	92.92
	5	2,752	20	85.34	86.21	85.34	84.24	85.33
		939	50	88.31	89.68	88.31	88.04	88.3
		429	100	89.67	90.28	89.67	89.56	89.65
		148	250	89.81	90.43	89.8	89.84	89.75
		117	300	89.14	89.47	89.14	89.04	89.06
		64	500	93.03	93.3	93.03	93.06	92.92
	6	2,752	20	86.12	86.75	86.12	85.97	86.12
		939	50	89.18	90.13	89.18	88.77	89.17
		429	100	90	90.58	90	89.82	89.98
		148	250	89.46	89.72	89.45	89.45	89.39
		117	300	89.94	90.35	89.94	89.95	89.86
		64	500	93.28	93.36	93.28	93.22	93.18
Genus	4	1,682	50	92.98	94.15	92.97	92.9	92.98
		1,293	100	94.78	95.24	94.77	94.74	94.78
		841	250	96.74	96.86	96.74	96.72	96.74
		580	500	97.59	97.65	97.58	97.59	97.58
	5	1,682	50	94.23	95.13	94.23	94.13	94.23
		1,293	100	95.37	95.35	95.36	95.35	95.33
		841	250	97	97.14	97	96.99	97
		580	500	97.54	97.59	97.54	97.54	97.54
	6	1,682	50	94.33	95.11	94.32	94.2	94.32
		1,293	100	95.39	95.76	95.39	95.35	95.39
		841	250	97.1	97.27	97.1	97.1	97.1
		580	500	97.83	97.88	97.83	97.83	97.83
Family	4	520	50	92.35	93.57	92.34	92.15	92.33
		453	100	95.32	95.9	95.32	95.32	95.31
		369	250	96.89	97.01	96.9	96.88	96.89
		301	500	97.37	97.42	97.37	97.37	97.37
	5	520	50	93.73	94.65	93.74	93.62	93.72
		453	100	96.11	96.54	96.11	96.08	96.11

		369	250	97.25	97.37	97.25	97.24	97.24
		301	500	97.82	97.86	97.83	97.81	97.81
	6	520	50	95.23	95.88	95.23	95.11	95.22
		453	100	96.64	96.94	96.64	96.63	96.64
		369	250	97.49	97.62	97.48	97.49	97.49
		301	500	98.21	98.24	98.2	98.2	98.2
Order	4	149	100	94.43	94.96	94.43	94.41	94.39
		106	500	98	98.05	98.01	98.01	97.98
	5	149	100	95.64	96.16	95.64	95.61	95.61
		106	500	98.33	98.39	98.34	98.34	98.32
	6	149	100	96.17	96.72	96.17	96.21	96.15
		106	500	98.19	98.25	98.19	98.19	98.17
Class	4	31	100	95.48	95.85	95.48	95.46	95.35
		25	500	98.32	98.39	98.31	98.32	98.25
	5	31	100	97.42	97.64	97.42	97.39	97.34
		25	500	98.88	98.9	98.87	98.87	98.83
	6	31	100	97.09	97.38	97.1	97.08	97.01
		25	500	98.8	98.86	98.8	98.8	98.75
Phylum	4	2	500	99	99.02	99	98.99	98.02
	5	2	500	100	100	100	100	100
	6	2	500	100	100	100	100	100
