# Supplementary material for: CNN_FunBar: Advanced Learning Technique for Fungi ITS Region Classification
Source: Genes (Basel). 2023 Mar 3;14(3):634. doi: 10.3390/genes14030634 (PMC10048311; doi:10.3390/genes14030634)
Supplement: Supplementary file 1 [file genes-14-00634-s001.zip › Table S2.pdf]

Table S2. Classification performances of CNN, SVM, KNN, Naïve-Bayes and Random Forest models for different k values at all taxonomic levels. Training datasets contain 100 datapoints (Class to Species) and 500 datapoints (Phylum) per category.

Taxonomic Level (N = No. of categories)	Model	*k*	Avg. Accuracy (%)	Avg. Precision (%)	Avg. Recall (%)	Avg. F1 Score (%)	Avg. MCC Score (%)
Species (N = 429)	CNN	4	89.14	89.78	89.13	89.01	89.11
		5	89.67	90.28	89.67	89.56	89.65
		6	90	90.58	90	89.82	89.98
	SVM	4	85.8	86.88	85.8	85.28	85.78
		5	87.35	88.69	87.35	86.96	87.33
		6	88.02	89.14	88.03	87.81	88.01
	KNN	4	86.71	87.54	86.7	86.48	86.69
		5	88.57	89.1	88.56	88.17	88.35
		6	88.58	89.11	88.58	88.21	88.5
	Naïve-Bayes	4	74.68	78.58	74.68	74.2	74.66
		5	75.17	78.43	75.18	75.16	75.17
		6	76.01	79.37	76.01	75.99	76.01
	RF	4	89.12	89.45	89.12	88.98	89.1
		5	89.34	89.45	89.34	89.17	89.31
		6	89.68	89.88	89.67	89.58	89.65
Genus (N = 1293)	CNN	4	94.78	95.24	94.77	94.74	94.78
		5	95.37	95.35	95.36	95.35	95.33
		6	95.39	95.76	95.39	95.35	95.39
	SVM	4	92.65	93.62	92.65	92.81	92.64
		5	92.98	93.49	92.98	92.78	92.95
		6	93.07	94.56	93.07	93.52	93.07
	KNN	4	93.1	93.76	93.1	92.96	93.08
		5	93.52	94.51	93.53	93.51	93.52
		6	93.64	94.66	93.64	93.71	93.63
	Naïve-Bayes	4	83.33	87.78	83.33	84.19	83.33
		5	84	84.12	84.01	83.89	83.98
		6	84.12	85.2	84.12	84.11	84.1
	RF	4	94.57	94.89	94.57	94.48	94.52
		5	95.01	95.29	95.01	94.99	95
		6	95.17	95.58	95.16	95.36	95.14
Family (N = 453)	CNN	4	95.32	95.9	95.32	95.32	95.31
		5	96.11	96.54	96.11	96.08	96.11
		6	96.64	96.94	96.64	96.63	96.64
	SVM	4	94.13	95.05	94.14	94.22	94.13
		5	95.22	96.34	95.21	95.42	95.21
		6	95.31	96.62	95.31	95.58	95.53

	KNN	4	93.07	93.85	93.06	92.98	93.05
		5	94.15	94.84	94.15	94.12	94.14
		6	93.71	94.82	93.72	93.13	93.71
	Naïve-Bayes	4	85.29	88.96	85.29	85.95	85.27
		5	85.79	87.64	85.78	85.69	85.77
		6	85.91	85.92	85.9	84.99	85.91
	RF	4	94.56	94.91	94.56	94.48	94.55
		5	95.58	95.95	95.57	95.56	95.57
		6	96.64	96.94	96.64	96.59	96.64
Order (N = 149)	CNN	4	94.43	94.96	94.43	94.41	94.39
		5	95.64	96.16	95.64	95.61	95.61
		6	96.17	96.72	96.17	96.21	96.15
	SVM	4	92.69	93.43	92.69	92.82	92.65
		5	94.02	94.87	94.01	94.19	93.98
		6	95.27	95.95	95.27	95.4	95.24
	KNN	4	91.54	92.43	91.54	91.44	91.49
		5	93.69	94.57	93.69	93.64	93.65
		6	93.73	95.22	93.74	93.76	93.71
	Naïve-Bayes	4	85.4	85.64	85.41	85.39	85.4
		5	87	87.58	87.01	87.47	87.01
		6	88.54	88.89	88.54	88.35	88.52
	RF	4	94.16	94.96	94.16	94.13	94.15
		5	95.63	95.14	95.63	95.59	95.6
		6	96.04	96.41	96.05	96.04	96.03
Class (N = 31)	CNN	4	95.48	95.85	95.48	95.46	95.35
		5	97.42	97.64	97.42	97.39	97.34
		6	97.09	97.38	97.1	97.08	97.01
	SVM	4	93.62	94.26	93.62	93.74	93.42
		5	95.19	95.75	95.19	95.32	95.05
		6	95.84	96.34	95.84	95.95	95.71
	KNN	4	92.52	93.22	92.52	92.46	92.36
		5	93.44	94.15	93.54	93.39	93.36
		6	92.11	95.59	92.11	92.33	92.01
	Naïve-Bayes	4	88.38	89.76	88.38	88.57	88.04
		5	89.16	89.26	89.16	89.2	89.15
		6	89.29	89.43	89.28	89.19	89.25
	RF	4	95.45	95.5	95.44	95.41	95.44
		5	97.06	97.26	97.06	97.05	97.01
		6	97.38	97.55	97.38	97.37	97.34
Phylum (N = 2)	CNN	4	99	99.02	99	98.99	98.02
		5	100	100	100	100	100
		6	100	100	100	100	100
	SVM	4	98.88	98.88	98.89	98.88	97.78

		5	99.55	99.56	99.55	99.55	99.1
		6	99.55	99.56	99.55	99.55	99.11
	KNN	4	99	99.01	99	98.99	99.01
		5	99.01	99.12	99.01	98.98	99.01
		6	100	100	100	100	100
	Naïve-Bayes	4	95	95.16	95	94.99	90.16
		5	96	96.15	96	95.98	93.78
		6	96.52	96.68	96.52	96.49	94.82
	RF	4	99	99.01	99	98.99	98.01
		5	99	99.02	99	98.99	99.02
		6	100	100	100	100	100
